# Supplementary figures and images for: The evolution and structure of snake venom phosphodiesterase (svPDE) highlight its importance in venom actions
Source: eLife. 2023 Apr 17;12:e83966. doi: 10.7554/eLife.83966 (PMC10121219; doi:10.7554/eLife.83966)

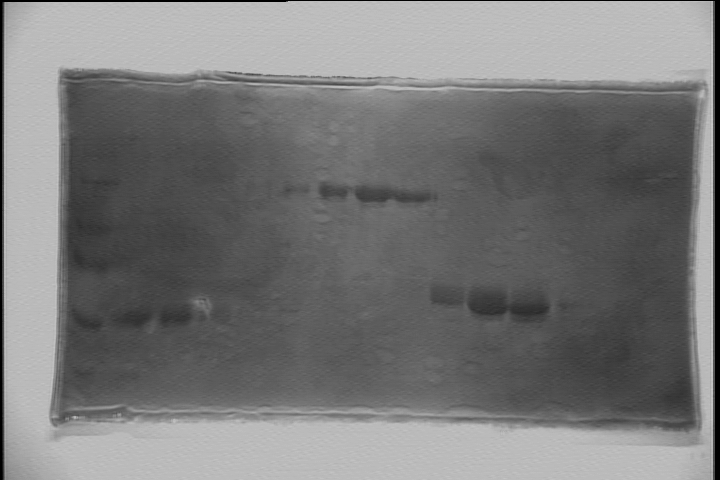

Supplement: Figure 3—source data 1. [file elife-83966-fig3-data1.zip › Souce_Data/Figure 3-figure supplement 1-1.tiff]

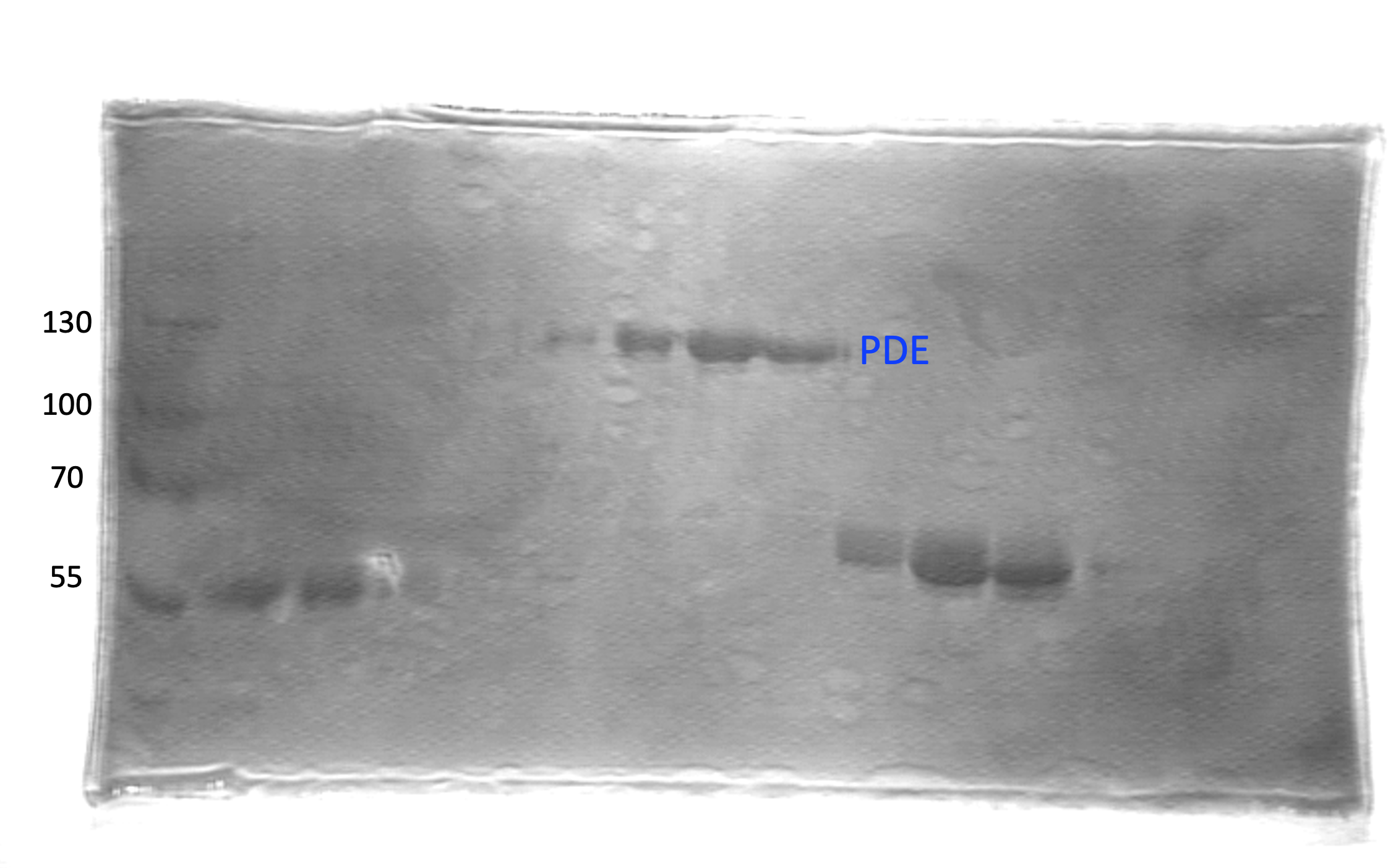

Supplement: Figure 3—source data 1. [file elife-83966-fig3-data1.zip › Souce_Data/Figure 3-figure supplement 1-2.png]
