# Supplementary material for: The evolution and structure of snake venom phosphodiesterase (svPDE) highlight its importance in venom actions
Source: eLife. 2023 Apr 17;12:e83966. doi: 10.7554/eLife.83966 (PMC10121219; doi:10.7554/eLife.83966)
Supplement: Supplementary file 1. [file elife-83966-supp1.docx]

**Supplementary File 1**

**Summary of genomic and transcriptomic data used in this study**

| **Family** | **NCBI accession** | **Data type** | **Scientific name** | **Common name** | **Source Tissue for RNA-Seq** |
| --- | --- | --- | --- | --- | --- |
| Shinisauridae | GCA_021292165.1 | Genome | *Shinisaurus crocodilurus* | Chinese crocodile lizard |  |
| Shinisauridae | SRR14583706 | RNA-Seq | *Shinisaurus crocodilurus* | Chinese crocodile lizard | Mixture |
| Varanidae | GCF_004798865.1 | Genome | *Varanus komodoensis* | Komodo dragon |  |
| Varanidae | SRR8466822 | RNA-Seq | *Varanus komodoensis* | Komodo dragon | blood |
| Varanidae | SRR8466824 | RNA-Seq | *Varanus komodoensis* | Komodo dragon | blood |
| Varanidae | SRR8466825 | RNA-Seq | *Varanus komodoensis* | Komodo dragon | blood |
| Varanidae | SRR8466826 | RNA-Seq | *Varanus komodoensis* | Komodo dragon | blood |
| Varanidae | SRR8466827 | RNA-Seq | *Varanus komodoensis* | Komodo dragon | blood |
| Varanidae | SRR8735151 | RNA-Seq | *Varanus komodoensis* | Komodo dragon | Heart |
| Varanidae | SRR8735152 | RNA-Seq | *Varanus komodoensis* | Komodo dragon | Heart |
| Agamidae | GCF_900067755.1 | Genome | *Pogona vitticeps* | Central bearded dragon |  |
| Agamidae | SRR14455628 | RNA-Seq | *Pogona vitticeps* | Central bearded dragon | Epithelium from dental tissues |
| Agamidae | SRR14455629 | RNA-Seq | *Pogona vitticeps* | Central bearded dragon | Epithelium from dental tissues |
| Agamidae | SRR14455630 | RNA-Seq | *Pogona vitticeps* | Central bearded dragon | Epithelium from dental tissues |
| Agamidae | SRR14455631 | RNA-Seq | *Pogona vitticeps* | Central bearded dragon | Mesenchyme from dental tissues |
| Agamidae | SRR14455632 | RNA-Seq | *Pogona vitticeps* | Central bearded dragon | Mesenchyme from dental tissues |
| Agamidae | SRR14455633 | RNA-Seq | *Pogona vitticeps* | Central bearded dragon | Mesenchyme from dental tissues |
| Agamidae | SRR14455634 | RNA-Seq | *Pogona vitticeps* | Central bearded dragon | Epithelium from dental tissues |
| Agamidae | SRR14455635 | RNA-Seq | *Pogona vitticeps* | Central bearded dragon | Epithelium from dental tissues |
| Agamidae | SRR14455636 | RNA-Seq | *Pogona vitticeps* | Central bearded dragon | Epithelium from dental tissues |
| Agamidae | SRR14455637 | RNA-Seq | *Pogona vitticeps* | Central bearded dragon | Mesenchyme from dental tissues |
| Agamidae | SRR14455638 | RNA-Seq | *Pogona vitticeps* | Central bearded dragon | Mesenchyme from dental tissues |
| Agamidae | SRR14455639 | RNA-Seq | *Pogona vitticeps* | Central bearded dragon | Mesenchyme from dental tissues |
| Dactyloidae | GCF_000090745.1 | Genome | *Anolis carolinensis* | Green anole |  |
| Dactyloidae | SRR495265 | RNA-Seq | *Anolis carolinensis* | Green anole | Adrenal Gland |
| Dactyloidae | SRR495266 | RNA-Seq | *Anolis carolinensis* | Green anole | Adrenal Gland |
| Dactyloidae | SRR495267 | RNA-Seq | *Anolis carolinensis* | Green anole | Adrenal Gland |
| Dactyloidae | SRR492481 | RNA-Seq | *Anolis carolinensis* | Green anole | Adrenal Gland |
| Dactyloidae | SRR492482 | RNA-Seq | *Anolis carolinensis* | Green anole | Adrenal Gland |
| Typhlopidae | GCA_022379055.1 | Genome | *Anilios bituberculatus* | Prong-snouted blind snake |  |
| Typhlopidae | SRR15431541 | RNA-Seq | unclassified *Madatyphlops* |  | Mixture |
| Pythonidae | GCF_000186305.1 | Genome | *Python bivittatus* | Burmese python |  |
| Pythonidae | SRR11149661 | RNA-Seq | *Python molurus* | Pythons | Spleen |
| Pythonidae | SRR11149662 | RNA-Seq | *Python molurus* | Pythons | Skeletal muscle |
| Pythonidae | SRR11149663 | RNA-Seq | *Python molurus* | Pythons | Brain |
| Pythonidae | SRR11149664 | RNA-Seq | *Python molurus* | Pythons | Ovary |
| Pythonidae | SRR11149665 | RNA-Seq | *Python molurus* | Pythons | Testis |
| Viperidae | GCA_003402635.1 | Genome | *Protobothrops flavoviridis* | Habu |  |
| Viperidae | DRR125541 | RNA-Seq | *Protobothrops flavoviridis* | Habu | Fang forming tissue |
| Viperidae | DRR125542 | RNA-Seq | *Protobothrops flavoviridis* | Habu | Venom gland |
| Viperidae | DRR125543 | RNA-Seq | *Protobothrops flavoviridis* | Habu | Pit organ |
| Viperidae | DRR125544 | RNA-Seq | *Protobothrops flavoviridis* | Habu | Nose |
| Viperidae | DRR125545 | RNA-Seq | *Protobothrops flavoviridis* | Habu | Brain |
| Viperidae | DRR125546 | RNA-Seq | *Protobothrops flavoviridis* | Habu | Eye |
| Viperidae | DRR125547 | RNA-Seq | *Protobothrops flavoviridis* | Habu | Fetal fibroblast |
| Viperidae | DRR125548 | RNA-Seq | *Protobothrops flavoviridis* | Habu | Venom gland |
| Viperidae | DRR125549 | RNA-Seq | *Protobothrops flavoviridis* | Habu | Brain |
| Viperidae | DRR125550 | RNA-Seq | *Protobothrops flavoviridis* | Habu | Spleen |
| Viperidae | DRR125551 | RNA-Seq | *Protobothrops flavoviridis* | Habu | Lung |
| Viperidae | DRR125552 | RNA-Seq | *Protobothrops flavoviridis* | Habu | Liver |
| Viperidae | DRR125553 | RNA-Seq | *Protobothrops flavoviridis* | Habu | Kidney |
| Viperidae | DRR125554 | RNA-Seq | *Protobothrops flavoviridis* | Habu | Pancreas |
| Viperidae | DRR125555 | RNA-Seq | *Protobothrops flavoviridis* | Habu | Small Intestine |
| Viperidae | DRR125556 | RNA-Seq | *Protobothrops flavoviridis* | Habu | Colon |
| Viperidae | DRR125557 | RNA-Seq | *Protobothrops flavoviridis* | Habu | Stomach |
| Viperidae | DRR125558 | RNA-Seq | *Protobothrops flavoviridis* | Habu | Heart |
| Viperidae | DRR125559 | RNA-Seq | *Protobothrops flavoviridis* | Habu | Ovary |
| Viperidae | DRR125560 | RNA-Seq | *Protobothrops flavoviridis* | Habu | Massseter muscle |
| Viperidae | GCA_018340635.1 | Genome | *Bothrops jararaca* | Jararaca |  |
| Viperidae | GCF_001527695.2 | Genome | *Protobothrops mucrosquamatus* | Taiwanese habu |  |
| Viperidae | GCA_003400415.2 | Genome | *Crotalus viridis* | Prairie rattlesnake |  |
| Viperidae | SRR7401978 | RNA-Seq | *Crotalus viridis* | Prairie rattlesnake | Liver |
| Viperidae | SRR7401979 | RNA-Seq | *Crotalus viridis* | Prairie rattlesnake | Liver |
| Viperidae | SRR7401980 | RNA-Seq | *Crotalus viridis* | Prairie rattlesnake | Kidney |
| Viperidae | SRR7401981 | RNA-Seq | *Crotalus viridis* | Prairie rattlesnake | Kidney |
| Viperidae | SRR7401982 | RNA-Seq | *Crotalus viridis* | Prairie rattlesnake | Liver |
| Viperidae | SRR7401983 | RNA-Seq | *Crotalus viridis* | Prairie rattlesnake | Liver |
| Viperidae | SRR7401984 | RNA-Seq | *Crotalus viridis* | Prairie rattlesnake | Kidney |
| Viperidae | SRR7401985 | RNA-Seq | *Crotalus viridis* | Prairie rattlesnake | Kidney |
| Viperidae | SRR7401986 | RNA-Seq | *Crotalus viridis* | Prairie rattlesnake | Pancreas |
| Viperidae | SRR7401987 | RNA-Seq | *Crotalus viridis* | Prairie rattlesnake | Tongue |
| Viperidae | SRR7401988 | RNA-Seq | *Crotalus viridis* | Prairie rattlesnake | Lung |
| Viperidae | SRR7401989 | RNA-Seq | *Crotalus viridis* | Prairie rattlesnake | Venom gland |
| Viperidae | SRR7401990 | RNA-Seq | *Crotalus viridis* | Prairie rattlesnake | Accessory venom gland |
| Viperidae | SRR7401991 | RNA-Seq | *Crotalus viridis* | Prairie rattlesnake | Testis |
| Viperidae | SRR7401992 | RNA-Seq | *Crotalus viridis* | Prairie rattlesnake | Pancreas |
| Viperidae | SRR7401993 | RNA-Seq | *Crotalus viridis* | Prairie rattlesnake | Shaker muscle |
| Viperidae | SRR7401994 | RNA-Seq | *Crotalus viridis* | Prairie rattlesnake | Stomach |
| Viperidae | SRR7401995 | RNA-Seq | *Crotalus viridis* | Prairie rattlesnake | Brain |
| Viperidae | SRR7401996 | RNA-Seq | *Crotalus viridis* | Prairie rattlesnake | Rictal gland |
| Viperidae | SRR7401997 | RNA-Seq | *Crotalus viridis* | Prairie rattlesnake | Ovary |
| Viperidae | SRR7401998 | RNA-Seq | *Crotalus viridis* | Prairie rattlesnake | Spleen |
| Viperidae | SRR7401999 | RNA-Seq | *Crotalus viridis* | Prairie rattlesnake | Blood |
| Viperidae | SRR7402000 | RNA-Seq | *Crotalus viridis* | Prairie rattlesnake | Liver |
| Viperidae | SRR7402001 | RNA-Seq | *Crotalus viridis* | Prairie rattlesnake | Kidney |
| Viperidae | SRR7402002 | RNA-Seq | *Crotalus viridis* | Prairie rattlesnake | Kidney |
| Viperidae | SRR7402003 | RNA-Seq | *Crotalus viridis* | Prairie rattlesnake | Kidney |
| Viperidae | SRR7402004 | RNA-Seq | *Crotalus viridis* | Prairie rattlesnake | Venom gland |
| Viperidae | SRR7402005 | RNA-Seq | *Crotalus viridis* | Prairie rattlesnake | Venom gland |
| Viperidae | SRR7402006 | RNA-Seq | *Crotalus viridis* | Prairie rattlesnake | Liver |
| Viperidae | SRR7402007 | RNA-Seq | *Crotalus viridis* | Prairie rattlesnake | Liver |
| Viperidae | SRR7402008 | RNA-Seq | *Crotalus viridis* | Prairie rattlesnake | Skin |
| Viperidae | SRR7402009 | RNA-Seq | *Crotalus viridis* | Prairie rattlesnake | Pancreas |
| Viperidae | GCF_016545835.1 | Genome | *Crotalus tigris* | Tiger rattlesnake |  |
| Homalopsidae | GCA_017656035.1 | Genome | *Myanophis thanlyinensis* |  |  |
| Homalopsidae | SRR12802475 | RNA-Seq | *Homalopsis buccata* | Masked water snake | Venom gland |
| Colubridae | GCF_001077635.1 | Genome | *Thamnophis sirtalis* | Common garter snake |  |
| Colubridae | SRR12915662 | RNA-Seq | *Thamnophis conanti* |  | Duvernoy's gland |
| Colubridae | SRR1292619 | RNA-Seq | *Boiga irregularis* |  | Duvernoy's gland |
| Colubridae | SRR14319402 | RNA-Seq | *Tantilla nigriceps* | Plain black-headed snake | Duvernoy's gland |
| Colubridae | SRR14319403 | RNA-Seq | *Tantilla nigriceps* | Plain black-headed snake | Duvernoy's gland |
| Colubridae | SRR14319404 | RNA-Seq | *Tantilla nigriceps* | Plain black-headed snake | Duvernoy's gland |
| Colubridae | SRR14319405 | RNA-Seq | *Tantilla nigriceps* | Plain black-headed snake | Duvernoy's gland |
| Colubridae | SRR14319406 | RNA-Seq | *Tantilla nigriceps* | Plain black-headed snake | Duvernoy's gland |
| Colubridae | SRR14319407 | RNA-Seq | *Tantilla nigriceps* | Plain black-headed snake | Duvernoy's gland |
| Colubridae | GCF_001185365.1 | Genome | *Pantherophis guttatus* | Corn snake |  |
| Colubridae | SRR9596700 | RNA-Seq | *Pantherophis guttatus* | Corn snake | Brain |
| Colubridae | SRR9596701 | RNA-Seq | *Pantherophis guttatus* | Corn snake | Brain |
| Colubridae | SRR9596706 | RNA-Seq | *Pantherophis guttatus* | Corn snake | Heart |
| Colubridae | SRR9596707 | RNA-Seq | *Pantherophis guttatus* | Corn snake | Liver |
| Colubridae | SRR9596708 | RNA-Seq | *Pantherophis guttatus* | Corn snake | Dorsal skin |
| Colubridae | SRR9596709 | RNA-Seq | *Pantherophis guttatus* | Corn snake | Ventral skin |
| Colubridae | SRR9596710 | RNA-Seq | *Pantherophis guttatus* | Corn snake | Vomeronasal Organ |
| Colubridae | SRR9596711 | RNA-Seq | *Pantherophis guttatus* | Corn snake | Vomeronasal Organ |
| Colubridae | SRR9596712 | RNA-Seq | *Pantherophis guttatus* | Corn snake | Cerebellum |
| Colubridae | SRR9596713 | RNA-Seq | *Pantherophis guttatus* | Corn snake | Testis |
| Colubridae | SRR9596714 | RNA-Seq | *Pantherophis guttatus* | Corn snake | Ovary |
| Colubridae | SRR9596715 | RNA-Seq | *Pantherophis guttatus* | Corn snake | Liver |
| Colubridae | SRR9596716 | RNA-Seq | *Pantherophis guttatus* | Corn snake | Kidney |
| Colubridae | SRR9596717 | RNA-Seq | *Pantherophis guttatus* | Corn snake | Cerebellum |
| Colubridae | GCA_019677565.1 | Genome | *Pituophis catenifer* | Copher snake |  |
| Colubridae | GCA_012654085.1 | Genome | *Pantherophis obsoletus* | Western rat snake |  |
| Colubridae | GCF_009769535.1 | Genome | *Thamnophis elegans* | Western terrestrial garter snake |  |
| Colubridae | SRR12802480 | RNA-Seq | *Rhabdophis subminiatus* | Red-necked keelback | Duvernoy's gland |
| Dipsadidae | GCA_003457575.1 | Genome | *Thermophis baileyi (Closed species to C. lineatus)* |  | Duvernoy's gland |
| Dipsadidae | SRR14319401 | RNA-Seq | *Conophis lineatus* |  | Duvernoy's gland |
| Dipsadidae | SRR12802479 | RNA-Seq | *Heterodon nasicus* | Western hognose snake | Duvernoy's gland |
| Dipsadidae | SRR12802481 | RNA-Seq | *Helicops leopardinus* |  | Duvernoy's gland |
| Dipsadidae | SRR1292610 | RNA-Seq | *Hypsiglena* |  | Duvernoy's gland |
| Lamprophiidae | SRR12802476 | RNA-Seq | *Psammophis subtaeniatus* | Western yellow-bellied sand snake | Venom gland |
| Lamprophiidae | SRR12802477 | RNA-Seq | *Psammophis schokari* | Schokari sand racer | Venom gland |
| Lamprophiidae | SRR12802478 | RNA-Seq | *Malpolon monspessulanus* | Montpellier snake | Venom gland |
| Elapidae | GCA_000516915.1 | Genome | *Ophiophagus hannah* | King cobra |  |
| Elapidae | GCA_015471245.1 | Genome | *Laticauda colubrina* | Yellow-lipped sea krait |  |
| Elapidae | GCF_900518725.1 | Genome | *Notechis scutatus* | Mainland tiger snake |  |
| Elapidae | SRR8206942 | RNA-Seq | *Naja haje* | Egyptian cobra | Venom gland |
| Elapidae | GCA_009733165.1 | Genome | *Naja naja* | Indian cobra |  |
| Elapidae | SRR8206943 | RNA-Seq | *Naja naja* | Indian cobra | Venom gland |
| Elapidae | SRR8206944 | RNA-Seq | *Naja kaouthia* | Monocled cobra | Venom gland |
| Elapidae | SRR11050878 | RNA-Seq | *Naja siamensis* | Thai spitting cobra | Venom gland |
| Elapidae | SRR11050880 | RNA-Seq | *Naja nubiae* | Nubian spitting cobra | Venom gland |
| Elapidae | SRR11050881 | RNA-Seq | *Naja nivea* | Cape cobra | Venom gland |
| Elapidae | This study | Genome | *Naja atra* | Chinese cobra | Muscle |
| Elapidae | SRR11050884 | RNA-Seq | *Naja atra* | Chinese cobra | Venom gland |
| Elapidae | SRR11050887 | RNA-Seq | *Naja sumatrana* | Equatorial spitting cobra | Venom gland |
| Elapidae | SRR11050889 | RNA-Seq | *Hemachatus haemachatus* | Rinkhals | Venom gland |
| Elapidae | SRR11050890 | RNA-Seq | *Naja philippinensis* | Philippine spitting cobra | Venom gland |
| Elapidae | SRR11050879 | RNA-Seq | *Naja pallida* | Red spitting cobra | Venom gland |
| Elapidae | SRR11050882 | RNA-Seq | *Naja nigricollis* | Black-necked spitting cobra | Venom gland |
| Elapidae | SRR11050883 | RNA-Seq | *Naja mossambica* | Mozambique spitting cobra | Venom gland |
| Elapidae | SRR11050885 | RNA-Seq | *Naja annulifera* | Snouted cobra | Venom gland |
| Elapidae | SRR11050886 | RNA-Seq | *Walterinnesia aegyptia* | Desert cobra | Venom gland |
| Elapidae | SRR11050888 | RNA-Seq | *Naja subfulva* | Brown forest cobra | Venom gland |
| Hydrophiidae | GCA_019472885.1 | Genome | *Hydrophis curtus* | Shaw’s sea snake |  |
| Hydrophiidae | SRR11659669 | RNA-Seq | *Hydrophis curtus* | Shaw’s sea snake | Venom gland |
| Hydrophiidae | SRR11659670 | RNA-Seq | *Hydrophis curtus* | Shaw’s sea snake | Venom gland |
| Hydrophiidae | SRR11659671 | RNA-Seq | *Hydrophis curtus* | Shaw’s sea snake | Venom gland |
| Hydrophiidae | GCA_019473425.1 | Genome | *Hydrophis cyanocinctus* | Blue-banded sea snake |  |
| Hydrophiidae | SRR11659657 | RNA-Seq | *Hydrophis cyanocinctus* | Blue-banded sea snake | Venom gland |
| Hydrophiidae | SRR11659658 | RNA-Seq | *Hydrophis cyanocinctus* | Blue-banded sea snake | Venom gland |
| Hydrophiidae | SRR11659659 | RNA-Seq | *Hydrophis cyanocinctus* | Blue-banded sea snake | Venom gland |
