# Supplementary material for: The evolution and structure of snake venom phosphodiesterase (svPDE) highlight its importance in venom actions
Source: eLife. 2023 Apr 17;12:e83966. doi: 10.7554/eLife.83966 (PMC10121219; doi:10.7554/eLife.83966)
Supplement: Supplementary file 2. — (A) Crystallographic and refinement statistics. (B) Direct biochemical measurement of the substrate specificity. [file elife-83966-supp2.docx]

**Supplementary File 2**

**A. Crystallographic and refinement statistics**

|  | ***Naja atra* svPDE** apo form (unliganded)  PDB accession: 5GZ4 | ***Naja atra* svPDE**  AMP-complexed form (liganded)  PDB accession: 5GZ5 |
| --- | --- | --- |
| **Data collection** |  |  |
| Wavelength (Å)  Space group | 0.97  P21212 | 1.00  P21212 |
| Cell dimensions |  |  |
| *a*, *b*, *c* (Å) | 171.679, 65.876, 89.615 | 171.207, 65.612, 88.675 |
| α, β, γ (°) | 90, 90, 90 | 90, 90, 90 |
| Resolution (Å) | 30 - 2.55 (2.64 – 2.55)* | 30 - 2.09 (2.18 – 2.09) |
| *R*_merge_ | 0.111 (0.560) | 0.059 (0.570) |
| CC_1/2_  *I*/σ*I* | 0.92 (0.723)  13.1 (2.0) | 0.945 (0.785)  22.5 (2.5) |
| Completeness (%) | 99.8 (99.8) | 99.1 (98.9) |
| Redundancy | 4.5 (4.7) | 4.8 (4.9) |
|  |  |  |
| **Refinement** |  |  |
| Resolution (Å) | 29.43 - 2.55 (2.64 – 2.55) | 27.88 - 2.09 (2.16 – 2.09) |
| No. of reflections  *R*_work/_ *R*_free_ | 33351  0.241/0.289 | 58940  0.181/0.217 |
| No. atoms |  |  |
| Protein  Glycan/ion  AMP | 6450  162 | 6255  157  23 |
| Water | 677 | 804 |
| B-factors |  |  |
| Protein  Glycan/ion  AMP | 49.39  52.42 | 37.91  56.27  39.12 |
| Water | 40.52 | 42.23 |
| R.m.s deviations |  |  |
| Bond lengths (Å) | 0.010 | 0.012 |
| Bond angles (º) | 1.78 | 1.52 |
| **Validation (MolProbity**^1^**)**  Ramachandran favored (%)  Ramachandran outliers (%) | 94.00  0.88 | 96.00  0.26 |

* Highest resolution shell is shown in parenthesis.

1. Chen, V.B., et al., *MolProbity: all-atom structure validation for macromolecular crystallography.* Acta Crystallogr D Biol Crystallogr, 2010. **66**(Pt 1): p. 12-21.

**B.** **Direct biochemical measurement of the substrate specificity**

| Protein | | **svPDE^*^** | **svPDE^2^** | **ENPP3^3^** | **ENPP1^4^** |
| --- | --- | --- | --- | --- | --- |
| Species | | *Naja atra* | *Trimeresurus stejneger* | *Homo sapiens* | *Homo sapiens* |
| Reaction Condition | | 37°C, pH8 | 37°C, pH7.4 | 37°C, pH7.5 | NA |
| ATP | K*_m_* (μM) | 336.0 | 360 ± 0.01 | 61.50 ± 6.40 | 144.5 ± 36.0 |
|  | k*_cat_* (s^-1^) | 44.0 | 2.68 ± 0.13 | 1.52 ± 0.41 | 7.8 ± 0.8 |
| ADP | K*_m_* (μM) | 654.0 | 91 ± 0.005 | NA | NA |
|  | k*_cat_* (s^-1^) | 37.0 | 0.23 ± 0.01 | NA | NA |
| NAD | K*_m_* (μM) | 539.0 | 240 ± 0.01 | NA | NA |
|  | k*_cat_* (s^-1^) | 608.0 | 0.82 ± 0.03 | NA | NA |
| GTP | K*_m_* (μM) | 2160.0 | NA | 123.70 ± 6.60 | NA |
|  | k*_cat_* (s^-1^) | 717.9 | NA | 3.37 ± 0.31 | NA |
| CTP | K*_m_* (μM) | 5750.0 | NA | 120.30 ± 10.50 | NA |
|  | k*_cat_* (s^-1^) | 1502.0 | NA | 6.36 ± 0.30 | NA |
| UTP | K*_m_* (μM) | 2730.0 | NA | 120.20 ± 26.80 | NA |
|  | k*_cat_* (s^-1^) | 684.6 | NA | 9.14 ± 0.68 | NA |

* This study.

2. Peng, L., et al., *Purification and partial characterization of a novel phosphodiesterase from the venom of Trimeresurus stejnegeri: inhibition of platelet aggregation.* Biochimie, 2011. **93**(9): p. 1601-9.

3. Gorelik, A., et al., *Structural basis for nucleotide recognition by the ectoenzyme CD203c.* FEBS J, 2018. **285**(13): p. 2481-2494.

4. Albright, R.A., et al., *Molecular basis of purinergic signal metabolism by ectonucleotide pyrophosphatase/phosphodiesterases 4 and 1 and implications in stroke.* J Biol Chem, 2014. **289**(6): p. 3294-306.
