# Supplementary material for: The evolution and structure of snake venom phosphodiesterase (svPDE) highlight its importance in venom actions
Source: eLife. 2023 Apr 17;12:e83966. doi: 10.7554/eLife.83966 (PMC10121219; doi:10.7554/eLife.83966)
Supplement: Supplementary file 3. — (A) Statistics of PacBio CCS reads. (B) Statistics of Illumina paired-end reads (C) Statistics of draft assembly. [file elife-83966-supp3.docx]

**Supplementary File 3**

**A.** **Statistics of PacBio CCS reads**

| **Library ID** | **Total pass bases (bp)** | **Total pass reads (#)** | **Mean length of pass reads (bp)** | **Max. length of pass reads (bp)** | **N50 length of pass reads (bp)** | **% reads**  **>10Kb** | **% reads**  **>20Kb** |
| --- | --- | --- | --- | --- | --- | --- | --- |
| m64144_201215_090635 | 29,676,086,054 | 2,375,903 | 12,490 | 39,549 | 12,666 | 87.91 | 0.50 |
| m64144_201216_153226 | 26,959,253,579 | 2,187,719 | 12,322 | 36,670 | 12,481 | 86.49 | 0.45 |
| Total | 56,635,339,633 | 4,563,622 | 12,410 | 39,549 | 12,578 | 87.23 | 0.48 |

**B.** **Statistics of Illumina paired-end reads**

| **Total reads** | **Clean reads** | **Total bases (bp)** | **Clean bases (bp)** | **Q20 (%)** | **Q30 (%)** | **GC (%)** |
| --- | --- | --- | --- | --- | --- | --- |
| 568,048,858 | 559,566,268 | 85,207,328,700 | 78,096,046,566 | 97.37 | 92.63 | 41.10 |

**C.** **Statistics of draft assembly**

| **Statistics** | **De novo assembly of PacBio reads** | | **Assembly polished with Illumina reads** | |
| --- | --- | --- | --- | --- |
|  | **Contig Length(bp)** | **Contig Number** | **Contig Length(bp)** | **Contig Number** |
| N50 | 29,710,036 | 18 | 29,709,188 | 18 |
| N60 | 20,075,995 | 26 | 20,074,295 | 26 |
| N70 | 10,560,158 | 38 | 10,559,546 | 38 |
| N80 | 4,204,041 | 65 | 4,203,549 | 65 |
| N90 | 1,530,490 | 140 | 1,530,247 | 140 |
| Longest | 111,070,803 | 1 | 111,070,038 | 1 |
| **Total** | 1,879,633,414 | 847 | 1,879,550,836 | 847 |
